# Device performances analysis of p-type doped silicene-based field effect transistor using SPICE-compatible model

**DOI:** 10.1371/journal.pone.0264483

**Published:** 2022-03-03

**Authors:** Mu Wen Chuan, Munawar Agus Riyadi, Afiq Hamzah, Nurul Ezaila Alias, Suhana Mohamed Sultan, Cheng Siong Lim, Michael Loong Peng Tan

**Affiliations:** 1 School of Electrical Engineering, Faculty of Engineering, Universiti Teknologi Malaysia, Skudai, Johor, Malaysia; 2 Department of Electrical Engineering, Diponegoro University, Semarang, Indonesia; University of Glasgow, UNITED KINGDOM

## Abstract

Moore’s Law is approaching its end as transistors are scaled down to tens or few atoms per device, researchers are actively seeking for alternative approaches to leverage more-than-Moore nanoelectronics. Substituting the channel material of a field-effect transistors (FET) with silicene is foreseen as a viable approach for future transistor applications. In this study, we proposed a SPICE-compatible model for p-type (Aluminium) uniformly doped silicene FET for digital switching applications. The performance of the proposed device is benchmarked with various low-dimensional FETs in terms of their on-to-off current ratio, subthreshold swing and drain-induced barrier lowering. The results show that the proposed p-type silicene FET is comparable to most of the selected low-dimensional FET models. With its decent performance, the proposed SPICE-compatible model should be extended to the circuit-level simulation and beyond in future work.

## 1. Introduction

In the modern lives, the computing power of digital devices has been improved by the technology innovations in the miniaturisation of semiconductor transistors [1]. The famous Moore’s Law will soon experience its fundamental limit because of various constraints in bulk silicon (Si) technology, especially in the sub-10-nm atomic scales [2–4]. Therefore, the more-than-Moore development of the alternative field-effect transistors (FETs) has attracted much attention in the nanoelectronic research communities. Numerous industrial and public funds and programmes were initiated globally to overcome these “roadblocks” for long-terms advances in computing technology beyond Moore’s Law [5].

Among the options in the more-than-Moore race, two-dimensional (2D) materials have emerged as the prospective contenders owing to their atomically thin structure. Following the success of graphene since 2004 [6], research activities regarding 2D materials are intensely stimulated. Until now, more than 1800 exfoliable 2D candidates are theoretically predicted based on density-functional theory (DFT) [7], among which silicene could play a major role in future transistors owing to its outstanding carrier mobility [8] and compatibility with the cutting-edge Si wafer technology [9]. Silicene was also shortlisted as a potential material for transistor miniaturisation in the International Roadmap for Devices and Systems (IRDS) [10].

In 2015, Tao *et al*. [9] fabricated the first silicene-based transistor operating at room temperature. Moreover, silicene nanosheets have successfully been fabricated on various substrates in their buckled [11–13] and planar [14] forms. However, the deposition of silicene monolayers on metals substrates is less practical viable for transistor applications, as compared to the direct growth on insulating layers, such as dielectrics or oxides [15]. This shortcoming can be addressed by using computational modelling and simulation while waiting for the breakthrough in silicene-based fabrication techniques.

Concerning the computational models of silicene-based transistors [16, 17], rigorous efforts were invested by many groups of researchers. The absence of bandgap in pristine silicene did not halt its exploration for nanotransistor applications. In spite of the challenges, various bandgap engineering techniques have been explored, and such examples include confinement through silicene nanoribbons (SiNRs) [18–20], co-decoration [21], and doping [22–24]. Among the aforementioned techniques, doping is the most commonly employed technique in the semiconductor industry to alter the electronic properties [25]. Furthermore, the performance of SiNR FETs are sensitive to their device dimensions [20, 26]; and it is still a major challenge to precisely control the widths of nanoribbons even for the established graphene monolayers [27]. Therefore, a uniformly aluminium (Al) doped silicene monolayer was proposed to engineer the bandgap of silicene, producing the AlSi_3_ monolayer. In addition, a SPICE-compatible model was created to facilitate studies beyond the device-level simulation [28], such as the gate and logic levels.

**Fig 1:**
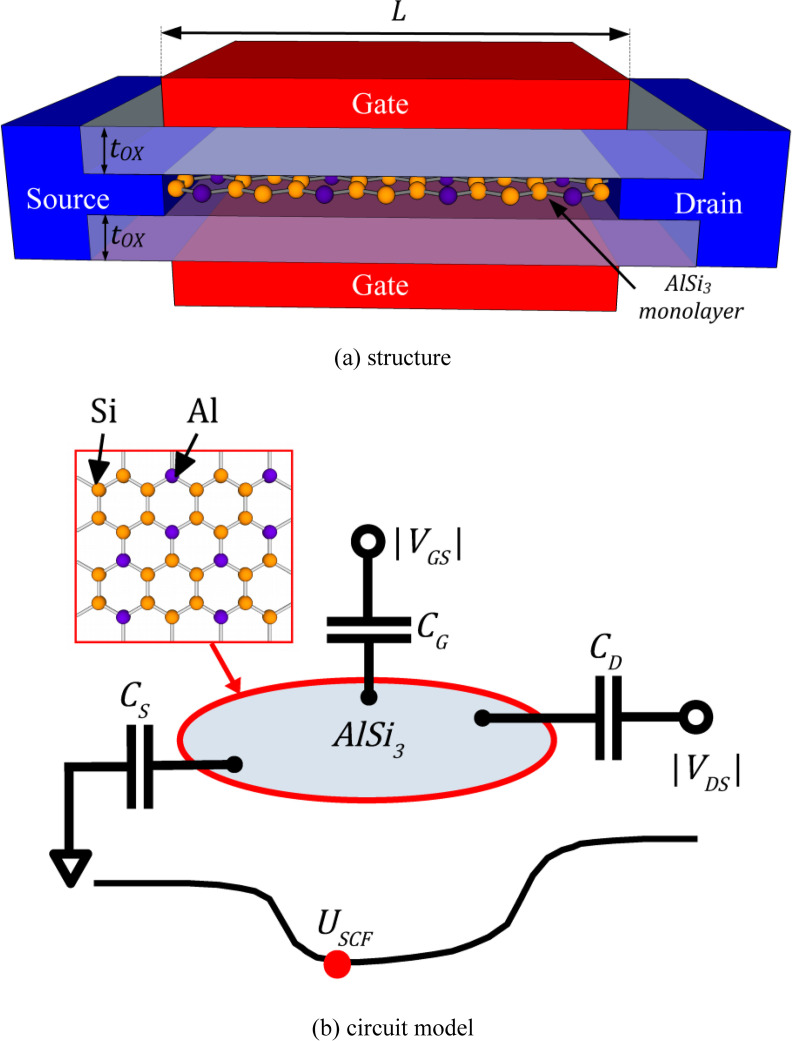
Schematic diagrams of AlSi_3_ FET: (a) the structure and (b) the ToB nanotransistor circuit model. The gate, drain and source terminal capacitances are denoted as *C*_G_, *C*_D_, and *C*_S_, respectively.

**Fig 1** shows the schematic diagrams of the proposed AlSi_3_ FET and the simplified top-of-the-barrier (ToB) nanotransistor circuit model. In this study, we developed of a SPICE-compatible model for the proposed AlSi_3_ FET from the ToB nanotransistor model and benchmarked its device performance metrics with other published low-dimensional transistor models. **Section 2** describes the modelling procedures to obtain the current-voltage (I-V) characteristics and the respective model evaluation methodology. **Section 3** discusses the device performance of AlSi_3_ FET with respect to its close low-dimensional contenders. Finally, **Section 4** includes the conclusion of this work and future work recommendation.

## 2. Methodology

**Fig 2:**
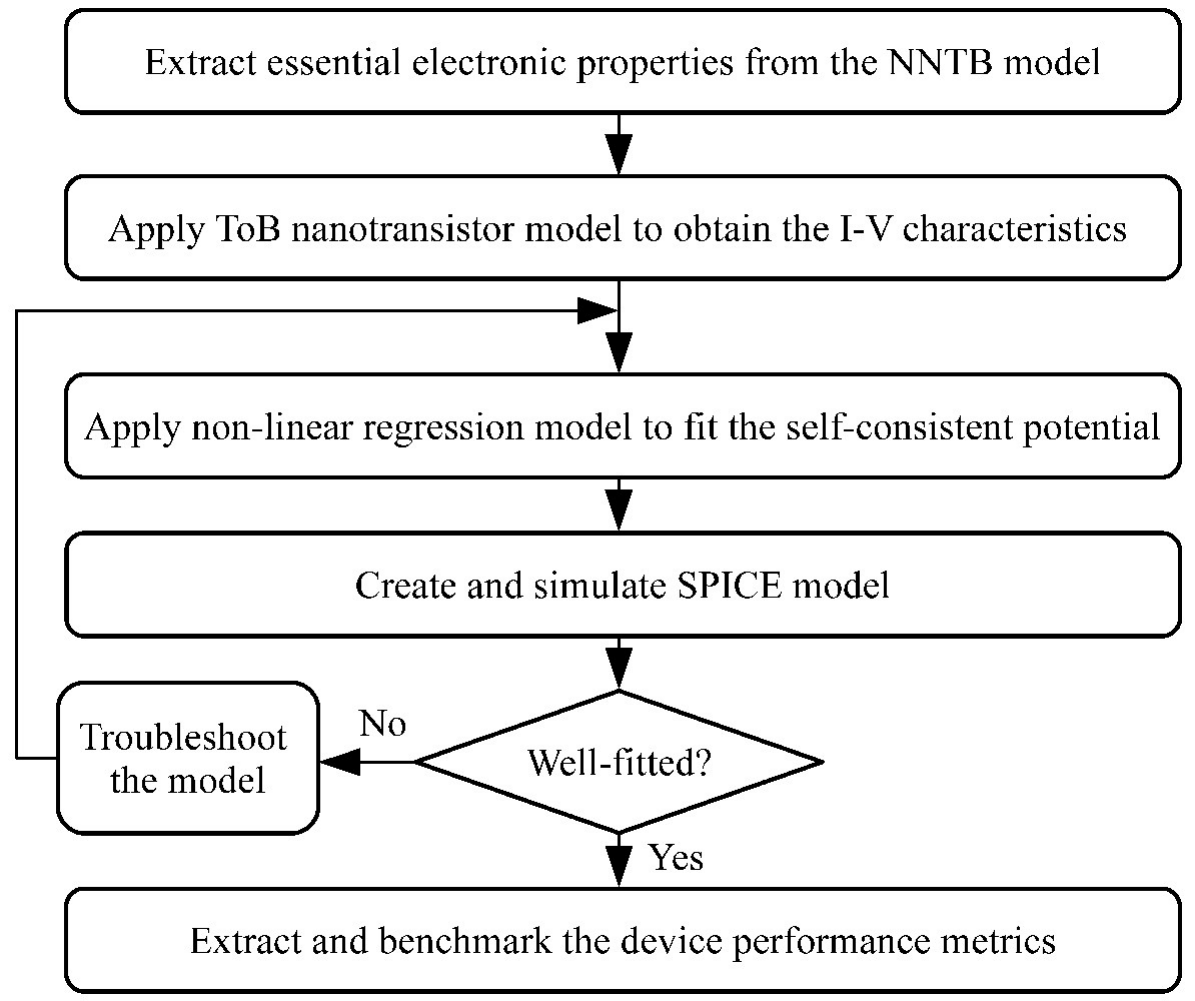
Overall flowchart of this study.

This section describes the overall modelling procedures employed in this study, where the overall flowchart is shown in **Fig 2**.

### 2.1. ToB nanotransistor and SPICE models

The atomic structure of the AlSi_3_ monolayer (as shown in **Fig 1**) was adapted from published DFT study [29]. By using the derivation from time-independent Schrödinger equation [30], the electronic transport effective mass was then obtained by using nearest neighbour tight-binding (NNTB) model and parabolic band assumptions as $m_{e}^{*}=0.235m_{0}$ and $m_{h}^{*}=0.255m_{0}$ for electrons and holes, respectively. The material-level modelling was shown in details in our previous work [22].

**Table 1** summarises the device parameters of the AlSi_3_ FETs. In the ToB nanotransistor model [31], net induced mobile charge can be obtained by Δ*P* = (*P*_*S*_+*P*_*D*_)−*P*_0_ where *P*_*S*_ and *P*_*D*_ are the non-equilibrium charge densities at the source and drain terminals, respectively, and *P*_0_ is the equilibrium charge density. The self-consistent potential *U*_*SCF*_ at the ToB is obtained by using

$$U_{SCF}=-q\left(\alpha_{G}|V_{GS}|+\alpha_{D}|V_{DS}|+\alpha_{S}V_{S}-q\frac{\Delta P}{C_{\Sigma }}\right),$$ (1)

where *q* is the constant for electric charge and the total terminal capacitances is expressed as *C*_Σ_ = *C*_G_+*C*_D_+*C*_S_. Because the source terminal is always set to be zero, *α*_*S*_ can be ignored. In an ideal FET, the perfect gate and drain control parameters *α*_*G*_ = 1 and *α*_*D*_ = 0 [18] are used to mimic the ideal I-V characteristics. However, the default gate and drain control parameters *α*_*G*_ = 0.880 and *α*_*D*_ = 0.035 [31] were used in this work.

**Table 1:** The device parameters of AlSi_3_ FETs.

Parameters	Values
Band structures	NNTB
Hole effective mass, $m_{h}^{*}$	0.255*m*_0_
Bandgap, *E*_*g*_	0.78 *eV*
Oxide material	SiO_2_
Oxide thickness, *t*_*OX*_	1.5 *nm*
Temperature, *T*	300 *K*

**Fig 3:**
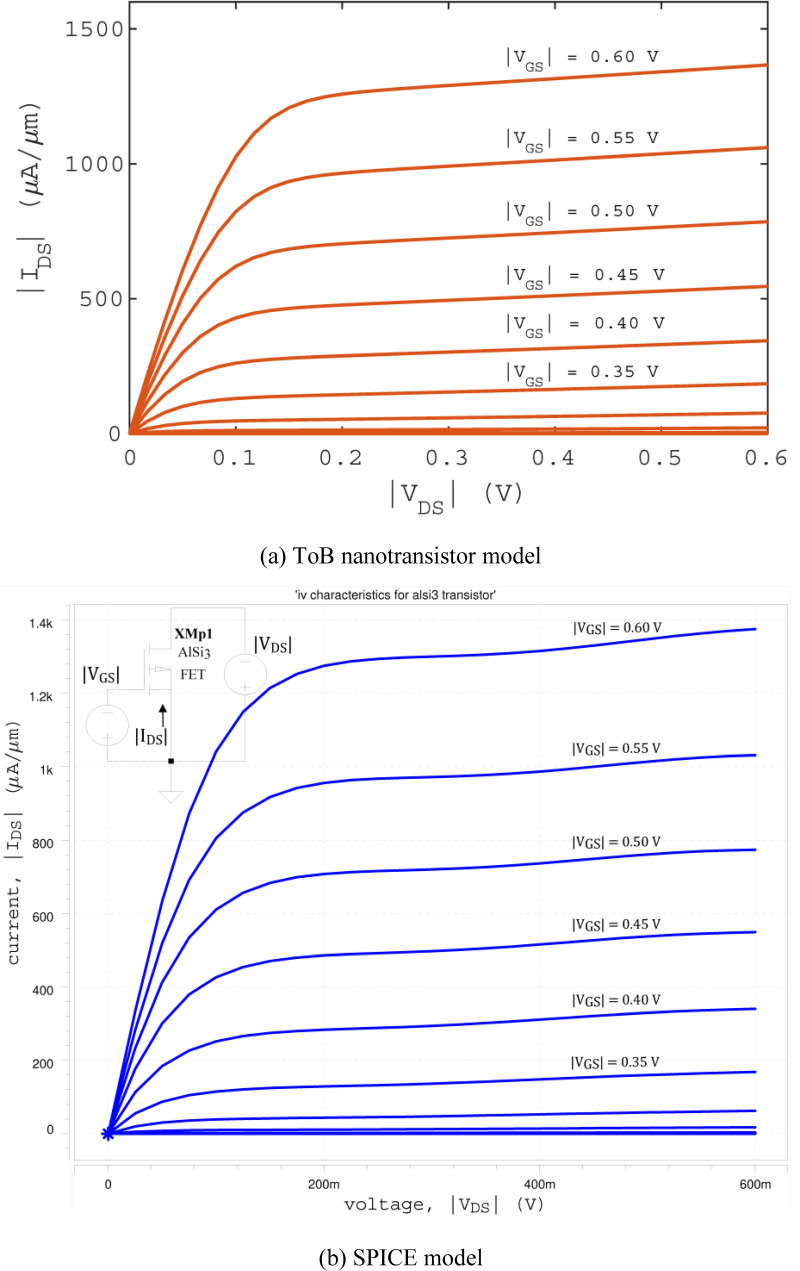
I-V characteristics of AlSi_3_ FET simulated using (a) ToB nanotransistor model and (b) SPICE model.

Subsequently, the current-voltage (I-V) characteristics of p-type AlSi_3_ FET can be obtained in terms of *V*_*DS*_ and *V*_*GS*_, by employing Landauer-Büttiker ballistic transport equation [31] along with Fermi-Dirac integral solutions [32], given as

$$|I_{DS}(\left|V_{GS}|,|V_{DS}\right|)|=\frac{gW}{\hbar^{2}}\sqrt{\frac{{m_{h}^{*}q^{2}(k_{B}T)}^{3}}{2\pi^{3}}}\{\log\left[1+e^{\eta_{S}(\left|V_{GS}|,|V_{DS}\right|)}\right]-\log\left[1+e^{\eta_{D}(\left|V_{GS}|,|V_{DS}\right|)}\right]\},$$ (2)

with the normalised energies of

$$\eta_{S}(\left|V_{GS}|,|V_{DS}\right|)=\frac{E_{F}-U_{SCF}(|V_{GS}|,|V_{DS}|)}{k_{B}T},$$ (3)

$$\eta_{D}(\left|V_{GS}|,|V_{DS}\right|)=\frac{E_{F}-U_{SCF}(|V_{GS}|,|V_{DS}|)-q|V_{DS}|}{k_{B}T},$$ (4)

for source and drain, respectively. In the equations, *g* is the degeneracy factor (set as 2 to include up and down spins); ℏ is the Planck’s constant; and *k*_*B*_ is the Boltzmann constant. **Fig 3(A)** shows the I-V characteristics for AlSi_3_ FET produced by the ToB nanotransistor model.

Following that, the results from ToB nanotransistor were further used to create the SPICE model to allow cross-platform and non-iterative simulation. Moreover, SPICE models are one of the essential tools in the IC design industry for simulation [28]. Before creating the SPICE model, a non-linear regression model [33] was employed to fit the self-consistent potential *U*_*SCF*_, given as

$$U_{SCF}(\left|V_{GS}|,|V_{DS}\right|)=\sum_{k=0\;to\;5}^{j=0\;to\;k}P_{\left\{j\}\{k-j\right\}}({\left|V_{GS}\right|}^{j}+{\left|V_{DS}\right|}^{k-j}),$$ (5)

where the coefficients *P*_{*j*}{*k*−*j*}_ for each respective |*V*_*GS*_|^*j*^|*V*_*DS*_|^*k*−*j*^ term were computed and optimised using MATLAB curve fitting tool. By using the MATLAB Curve Fitting tool, it was found that the lower orders of polynomial equations fail to fit the USCF well and, as a result, are unable to reproduce accurate I-V characteristics. Therefore, the fifth-order polynomial equation (highest number of order available in the MATLAB Curve Fitting tool) is chosen although the resulting equation is slightly long. The expansion of **Eq (5)** and the respective coefficients *P*_{*j*}{*k*−*j*}_ are attached along with the SPICE model library files in the S1 File. **Fig 3(B)** shows the I-V characteristics for AlSi_3_ FET produced by the SPICE model.

### 2.2. Model evaluation

**Fig 4:**
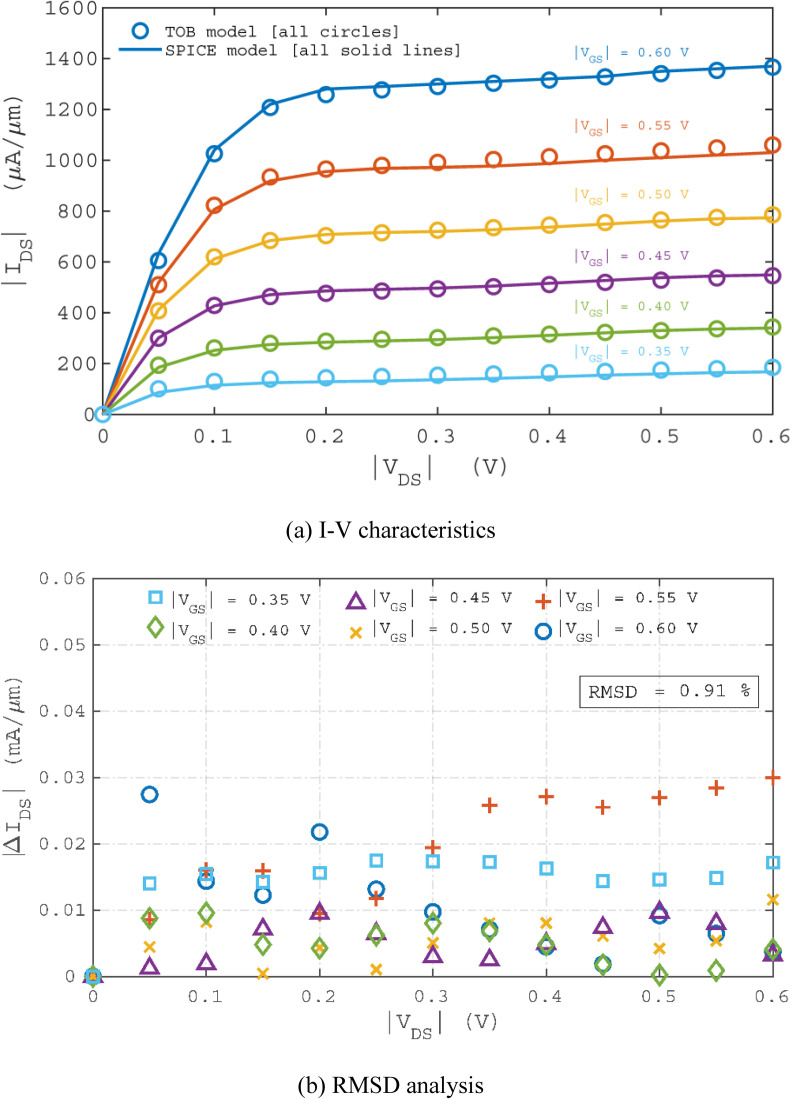
Comparison between the ToB nanotransistor model and SPICE model of the AlSi_3_ FET. The empty dots in (a) represent the results of the ToB nanotransistor model while the solid lines in (a) represent the results of the SPICE model.

In this subsection, a statistical method is employed to evaluate the accuracy of the SPICE model with respect to the ToB nanotransistor model for the proposed AlSi_3_ FET. The models were evaluated by using the normalised root-mean-square-deviations (RMSD) [29], given as

$$RMSD=\frac{\sqrt{\sum_{i=1}^{N}{\left(p_{i}-q_{i}\right)}^{2}/N}}{\max\left(p_{i},q_{i}\right)-min(p_{i},q_{i})}\times 100\%,$$ (6)

where *N* denotes the total number of data, *p*_*i*_ and *q*_*i*_ are the values of *i*^*th*^ data for the ToB nanotransistor model and the SPICE model, respectively. **Fig 4(A)** shows the I-V characteristics combining the results of the ToB nanotransistor model and the SPICE model. The point-by-point differences to compute RMSD are plotted in **Fig 4(B)**. Overall, 0.91% of RMSD is produced by the SPICE model when it is benchmarked with the results from the ToB nanotransistor model, indicating that the model has produced a decent fit.

## 3. Performances analysis and discussion

**Fig 5:**
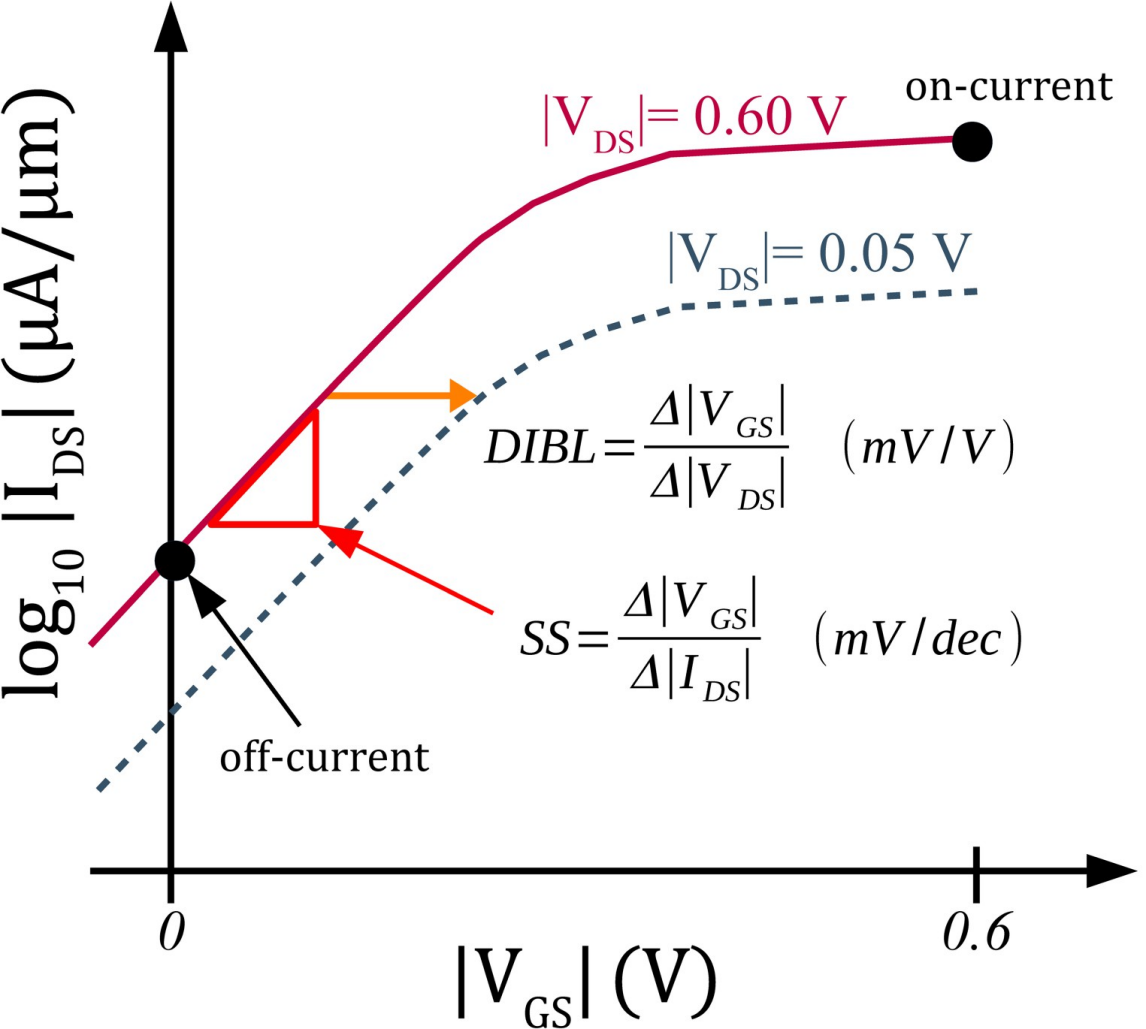
Definitions of the device performance metrics of a FET.

We can analyse the device performances by extracting the device metrics from the proposed p-type AlSi_3_ FET model. **Fig 5** shows the graphical extraction approach to obtain on-to-off current (*I*_*on*_/*I*_*off*_) ratio, subthreshold swing (SS), and drain-induced barrier lowering (DIBL). The proposed AlSi_3_ FET produces an *I*_*on*_/*I*_*off*_ ratio of 2.6×10^5^, a SS of 67.8 *mV*/*dec*, and a DIBL of 48.2 *mV*/*V*.

**Fig 6:**
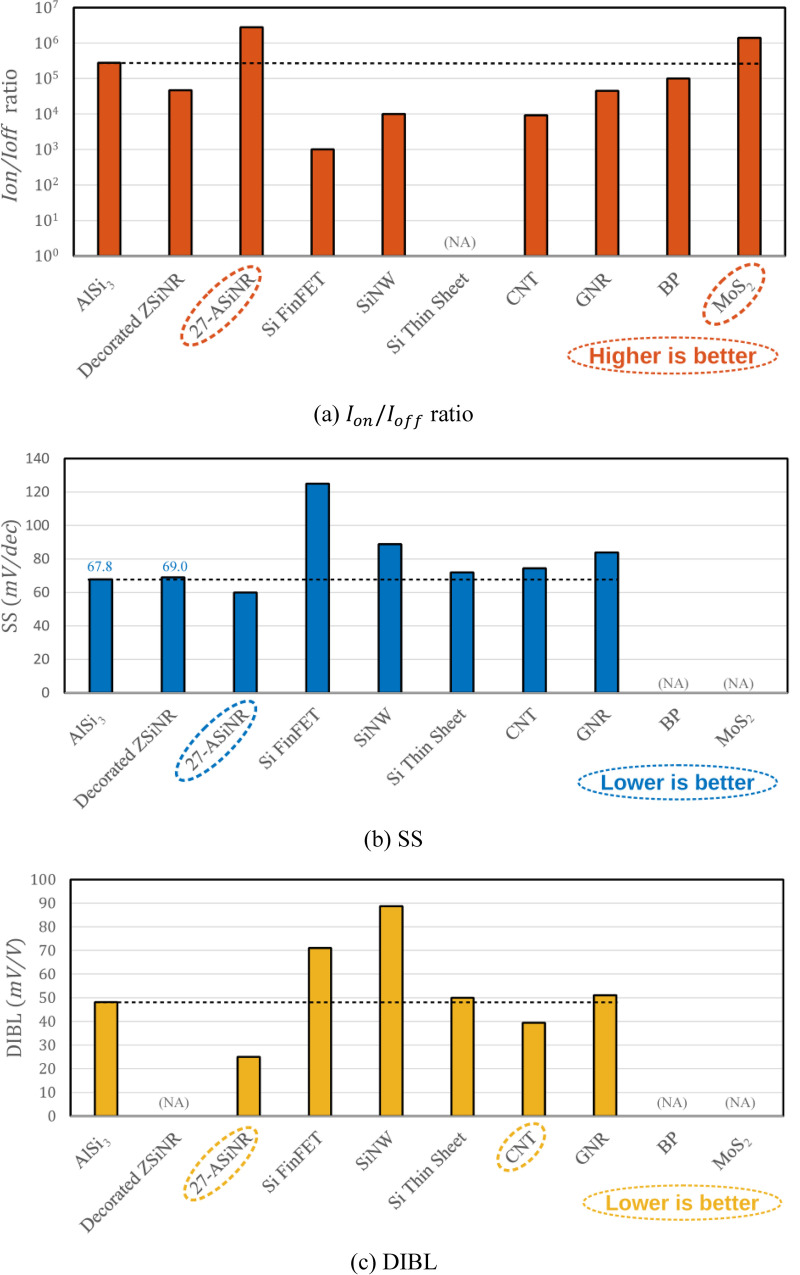
Device performances analysis between the proposed AlSi_3_ FET model with published transistor models based on various low-dimensional materials. NA (not available) in the bar graphs denotes the unavailable data.

In this work, we compared our proposed p-type AlSi_3_ FET with respect to other low-dimensional FETs. We have selected other published works on low-dimensional FETs to fairly assess the device performance of the proposed AlSi_3_ FET. The selected published models include co-decorated SiNR FET [21], 27-ASiNR FET [20], Si nanowire (SiNW) FET [34], Si thin sheet FET [35], carbon nanotube (CNT) FET [36], graphene nanoribbon (GNR) FET [37], black phosphorene (BP) FET [38], and monolayer molybdenum disulfide (MoS_2_) FET [39]. To concisely compare the device performance metrics, the comparisons are presented as bar graphs as shown in **Fig 6**. Regarding the *I*_*on*_/*I*_*off*_ ratio, the performance of AlSi_3_ FET model is slightly inferior to 27-ASiNR FET [20] and MoS_2_ FET [39]. Regarding the SS, AlSi_3_ FET model is also slightly higher than the 27-ASiNR FET [20]. Concerning the DIBL, AlSi_3_ FET model is also outperformed by 27-ASiNR FET [20] and CNT FET [36]. However, the challenges remain owing to the fabrication compatibility of non-Si-based materials and the difficulty to precisely control the widths of nanoribbons [27, 40]. Therefore, the proposed AlSi_3_ FET model is still a prospective alternative for future nanotransistor applications.

## 4. Conclusion

In this paper, we have investigated a SPICE-compatible model for p-type uniformly Al-doped silicene FET. Following that, the device performance of the proposed model is compared with other published low-dimensional nanotransistors. Although the proposed silicene FET is slightly inferior to a few competitors, silicene-based FETs are still one of the potential ways for more-than-Moore nanoelectronic applications owing to its Si-based nature. This work can be extended by performing further circuit-level simulation beyond the transistor device level by using the proposed SPICE model.

## Supporting information

S1 File SPICE model. (DOCX)
