# Supplementary material for: Device performances analysis of p-type doped silicene-based field effect transistor using SPICE-compatible model
Source: PLoS One. 2022 Mar 3;17(3):e0264483. doi: 10.1371/journal.pone.0264483 (PMC8893636; doi:10.1371/journal.pone.0264483)
Supplement: S1 File — (DOCX) [file pone.0264483.s001.docx]

**Device performances analysis of p-type doped silicene-based field effect transistor using SPICE-compatible model**

Mu Wen, CHUAN^1^; Munawar Agus, RIYADI^2^; Afiq, HAMZAH^1^; Nurul Ezaila, ALIAS^1^; Suhana, MOHAMED SULTAN^1^; Cheng Siong, LIM^1^; Michael Loong Peng, TAN^1*^

*^1^School of Electrical Engineering, Faculty of Engineering, Universiti Teknologi Malaysia, 81310 Skudai, Johor, Malaysia.*

*^2^Department of Electrical Engineering, Diponegoro University, Semarang 50275, Indonesia.*

**Corresponding email:* [*michael@utm.my*](mailto:michael@utm.my)

**ELECTRONIC SUPPLEMENTARY INFORMATION / APPENDIX**

A1. Expanding firth order binomial equation

Expand **Eqn.** **(5)**

$$U_{SCF}\left( \left| V_{GS} \right|,\left| V_{DS} \right| \right)=\sum_{k=0 to 5}^{j=0 to k} P_{\left\{ j \right\}\{k-j\}}(\left| V_{GS} \right|^{j}+\left| V_{DS} \right|^{k-j}) ,$$

to obtain

$$U_{SCF}\left( \left| V_{GS} \right|,\left| V_{DS} \right| \right)=P_{00}\left| V_{GS} \right|^{0}\left| V_{DS} \right|^{0}+P_{10}\left| V_{GS} \right|^{1}\left| V_{DS} \right|^{0}+P_{01}\left| V_{GS} \right|^{0}\left| V_{DS} \right|^{1}+P_{20}\left| V_{GS} \right|^{2}\left| V_{DS} \right|^{0}+P_{11}\left| V_{GS} \right|^{1}\left| V_{DS} \right|^{1}+P_{02}\left| V_{GS} \right|^{0}\left| V_{DS} \right|^{2}+P_{30}\left| V_{GS} \right|^{3}\left| V_{DS} \right|^{0}+P_{21}\left| V_{GS} \right|^{2}\left| V_{DS} \right|^{1}+P_{12}\left| V_{GS} \right|^{1}\left| V_{DS} \right|^{2}{+P}_{03}\left| V_{GS} \right|^{0}\left| V_{DS} \right|^{3}+P_{40}\left| V_{GS} \right|^{4}\left| V_{DS} \right|^{0}+P_{31}\left| V_{GS} \right|^{3}\left| V_{DS} \right|^{1}+P_{22}\left| V_{GS} \right|^{2}\left| V_{DS} \right|^{2}+P_{13}\left| V_{GS} \right|^{1}\left| V_{DS} \right|^{3}{+P}_{04}\left| V_{GS} \right|^{0}\left| V_{DS} \right|^{4}+P_{50}\left| V_{GS} \right|^{5}\left| V_{DS} \right|^{0}+P_{41}\left| V_{GS} \right|^{4}\left| V_{DS} \right|^{1}+P_{32}\left| V_{GS} \right|^{3}\left| V_{DS} \right|^{2}+P_{23}\left| V_{GS} \right|^{2}\left| V_{DS} \right|^{3}+P_{14}\left| V_{GS} \right|^{1}\left| V_{DS} \right|^{4}{+P}_{05}\left| V_{GS} \right|^{0}\left| V_{DS} \right|^{5} ,$$

After simplifying the equation, we obtain

$$U_{SCF}\left( \left| V_{GS} \right|,\left| V_{DS} \right| \right)=P_{00}+P_{10}\left| V_{GS} \right|+P_{01}\left| V_{DS} \right|+P_{20}\left| V_{GS} \right|^{2}+P_{11}\left| V_{GS} \right|\left| V_{DS} \right|+P_{02}\left| V_{DS} \right|^{2}+P_{30}\left| V_{GS} \right|^{3}+P_{21}\left| V_{GS} \right|^{2}\left| V_{DS} \right|+P_{12}\left| V_{GS} \right|\left| V_{DS} \right|^{2}{+P}_{03}\left| V_{DS} \right|^{3}+P_{40}\left| V_{GS} \right|^{4}+P_{31}\left| V_{GS} \right|^{3}\left| V_{DS} \right|+P_{22}\left| V_{GS} \right|^{2}\left| V_{DS} \right|^{2}+P_{13}\left| V_{GS} \right|\left| V_{DS} \right|^{3}{+P}_{04}\left| V_{DS} \right|^{4}+P_{50}\left| V_{GS} \right|^{5}+P_{41}\left| V_{GS} \right|^{4}\left| V_{DS} \right|+P_{32}\left| V_{GS} \right|^{3}\left| V_{DS} \right|^{2}+P_{23}\left| V_{GS} \right|^{2}\left| V_{DS} \right|^{3}+P_{14}\left| V_{GS} \right|\left| V_{DS} \right|^{4}{+P}_{05}\left| V_{DS} \right|^{5}.$$

A total of 21 terms; and therefore, 21 coefficients are produced after expanding the fifth order binomial equation. Following this, the coefficients $P_{\left\{ j \right\}\{k-j\}}$ for each respective $\left| V_{GS} \right|^{j}\left| V_{DS} \right|^{j-k}$ term were computed and optimised using MATLAB curve fitting tool.

A2. $\boldsymbol{U}_{\boldsymbol{SCF}}\left( \left| \boldsymbol{V}_{\boldsymbol{GS}} \right|\boldsymbol{,}\left| \boldsymbol{V}_{\boldsymbol{DS}} \right| \right)$ for p-type uniformly doped silicene (AlSi_3_) FET

Table S1. Values of the coefficients in the $\boldsymbol{U}_{\boldsymbol{SCF}}\left( \left| \boldsymbol{V}_{\boldsymbol{GS}} \right|\boldsymbol{,}\left| \boldsymbol{V}_{\boldsymbol{DS}} \right| \right)$ for AlSi_3_ FET.

| Coefficients | Values |  | Coefficients | Values |
| --- | --- | --- | --- | --- |
| $\boldsymbol{P}_{\boldsymbol{00}}$ | $\mathbf{+}\mathbf{0.020280}$ |  | $\boldsymbol{P}_{\boldsymbol{31}}$ | $\mathbf{-}\mathbf{0}\mathbf{.}\mathbf{786000}$ |
| $\boldsymbol{P}_{\boldsymbol{10}}$ | $\mathbf{-}\mathbf{0}\mathbf{.}\mathbf{762400}$ |  | $\boldsymbol{P}_{\boldsymbol{22}}$ | $\mathbf{+}\mathbf{3}\mathbf{.}\mathbf{558000}$ |
| $\boldsymbol{P}_{\boldsymbol{01}}$ | $\boldsymbol{-}\boldsymbol{0}\boldsymbol{.}\boldsymbol{039390}$ |  | $\boldsymbol{P}_{\boldsymbol{13}}$ | $\mathbf{-}\mathbf{5}\mathbf{.}\mathbf{240000}$ |
| $\boldsymbol{P}_{\boldsymbol{20}}$ | $\mathbf{-}\mathbf{1}\mathbf{.}\mathbf{342000}$ |  | $\boldsymbol{P}_{\boldsymbol{04}}$ | $\mathbf{+}\mathbf{3}\mathbf{.}\mathbf{119000}$ |
| $\boldsymbol{P}_{\boldsymbol{11}}$ | $\mathbf{-}\mathbf{0}\mathbf{.}\mathbf{144800}$ |  | $\boldsymbol{P}_{\boldsymbol{50}}$ | $\mathbf{-}\mathbf{2}\mathbf{.}\mathbf{001000}$ |
| $\boldsymbol{P}_{\boldsymbol{02}}$ | $\mathbf{+}\mathbf{0}\mathbf{.}\mathbf{182200}$ |  | $\boldsymbol{P}_{\boldsymbol{41}}$ | $\mathbf{+}\mathbf{0}\mathbf{.}\mathbf{009736}$ |
| $\boldsymbol{P}_{\boldsymbol{30}}$ | $\mathbf{+}\mathbf{4}\mathbf{.}\mathbf{472000}$ |  | $\boldsymbol{P}_{\boldsymbol{32}}$ | $\mathbf{+}\mathbf{0}\mathbf{.}\mathbf{190900}$ |
| $\boldsymbol{P}_{\boldsymbol{21}}$ | $\mathbf{-}\mathbf{0}\mathbf{.}\mathbf{600900}$ |  | $\boldsymbol{P}_{\boldsymbol{23}}$ | $\mathbf{-}\mathbf{3}\mathbf{.}\mathbf{254000}$ |
| $\boldsymbol{P}_{\boldsymbol{12}}$ | $\mathbf{+}\mathbf{1}\mathbf{.}\mathbf{56300}$ |  | $\boldsymbol{P}_{\boldsymbol{14}}$ | $\mathbf{+}\mathbf{4}\mathbf{.}\mathbf{847000}$ |
| $\boldsymbol{P}_{\boldsymbol{03}}$ | $\mathbf{-}\mathbf{1}\mathbf{.}\mathbf{279000}$ |  | $\boldsymbol{P}_{\boldsymbol{05}}$ | $\mathbf{-}\mathbf{2}\mathbf{.}\mathbf{461000}$ |
| $\boldsymbol{P}_{\boldsymbol{40}}$ | $\mathbf{-}\mathbf{1}\mathbf{.}\mathbf{672000}$ |  | - | - |

B1. SPICE library file for parameter declaration (param.lib)


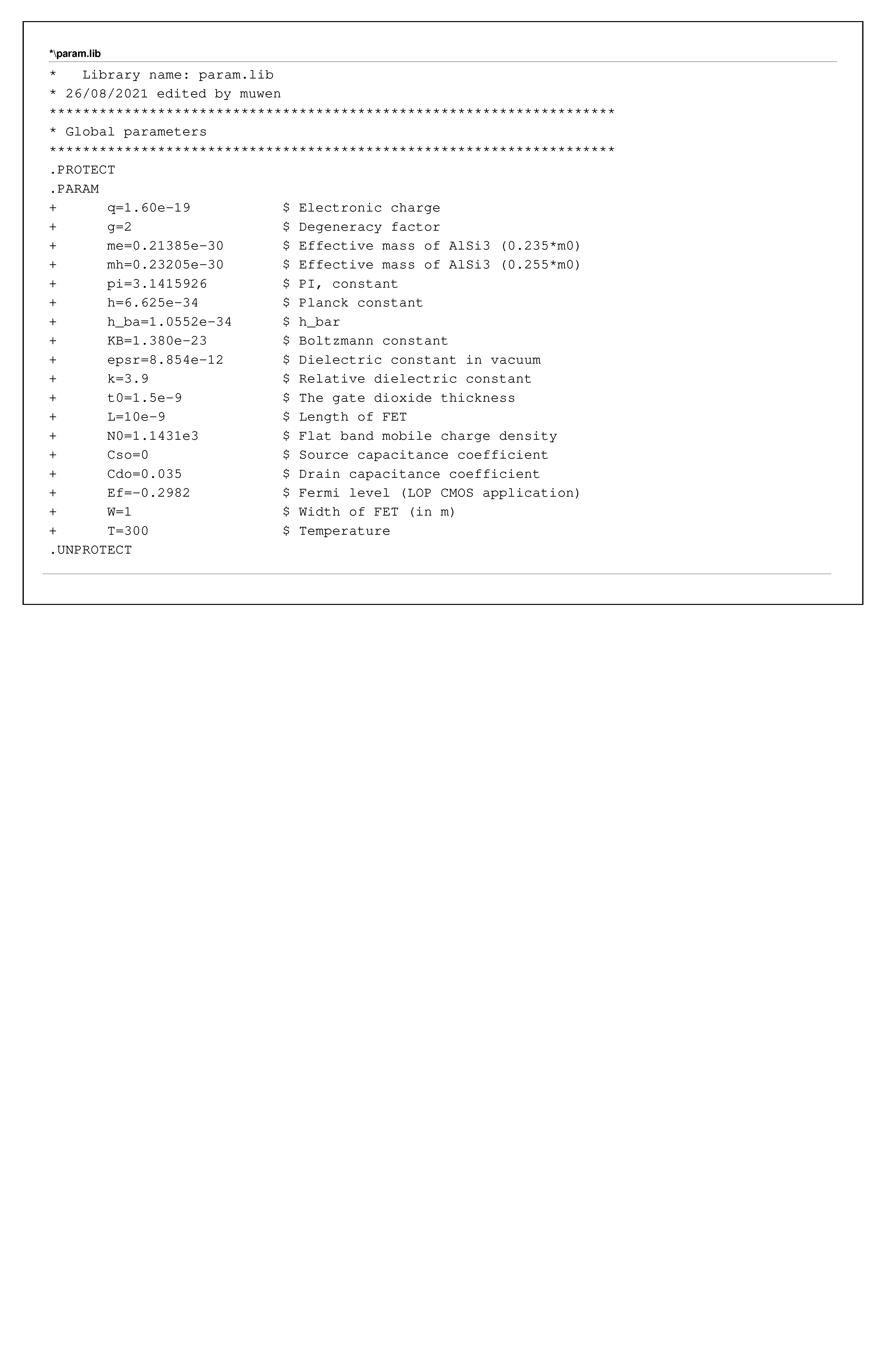


B2. SPICE library file for transistor model (CSmuwen.lib)


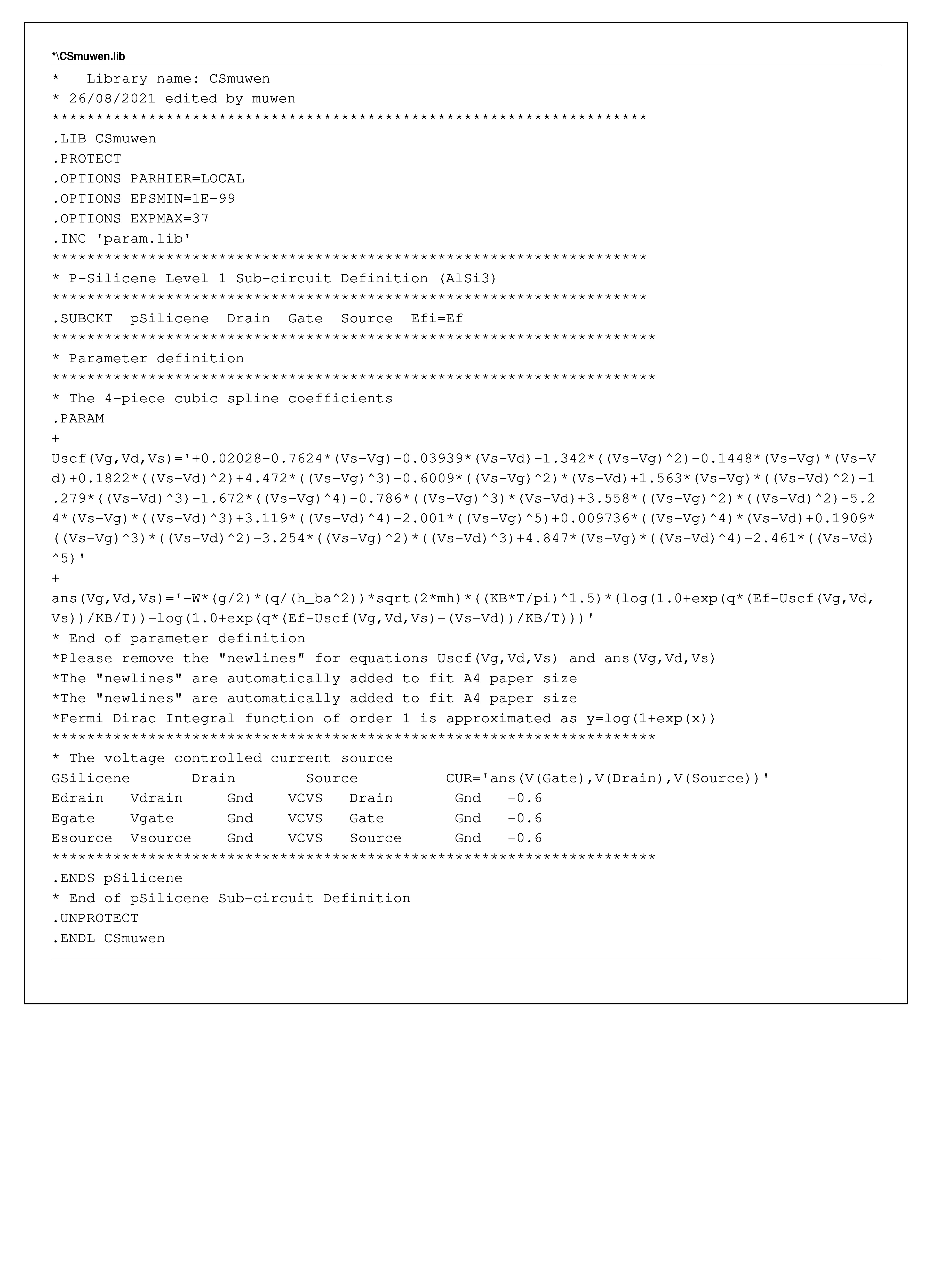


B3. SPICE file for transistor simulation (AlSi3FETmodel.sp)


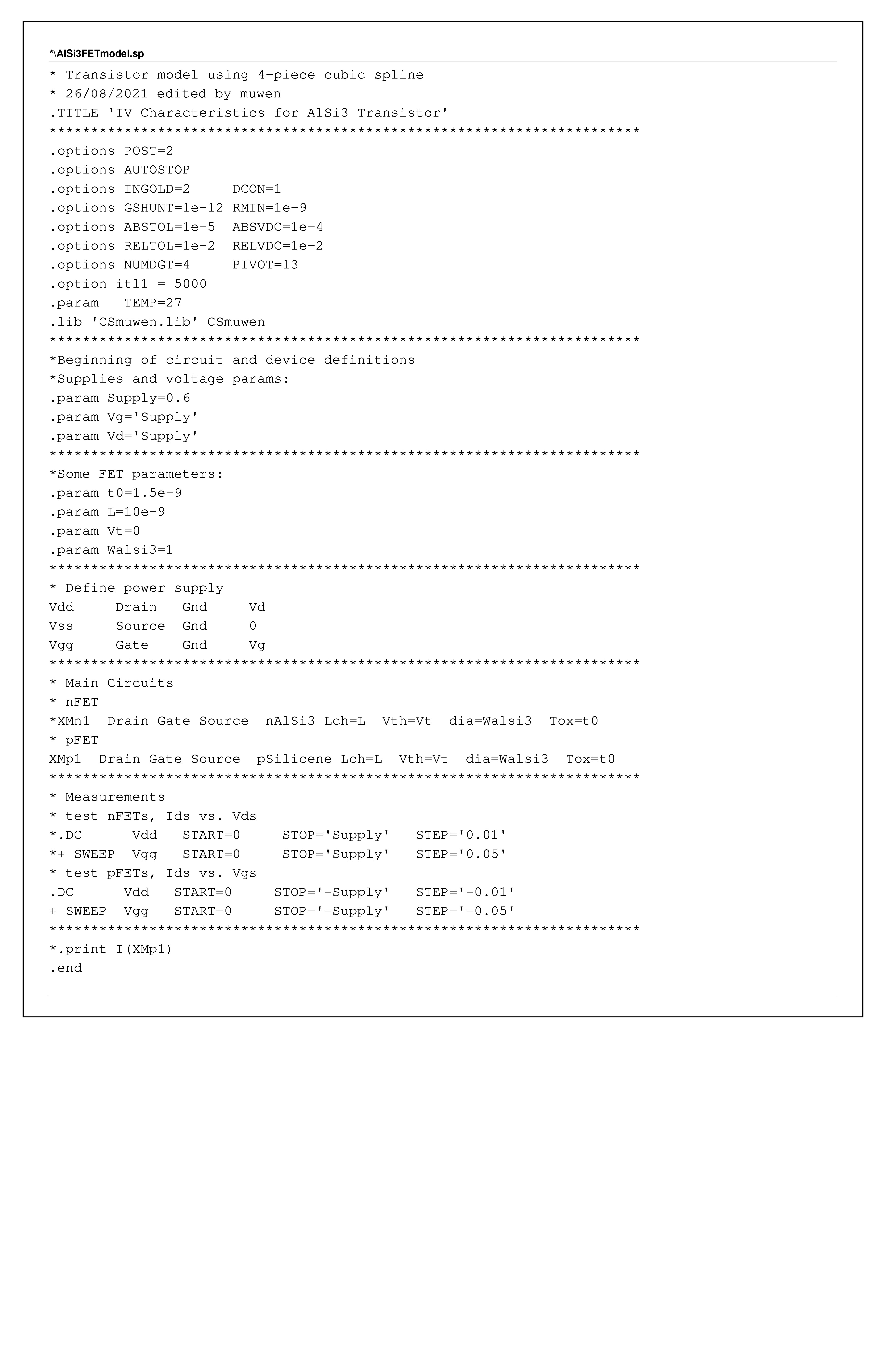


-----------------------------------------------------------------------------------------------------------------------------------

**-End of File-**
